# Changes in Cancer Screening Rates Following a New Cancer Diagnosis in a Primary Care Patient Panel

**DOI:** 10.1001/jamanetworkopen.2022.22131

**Published:** 2022-07-15

**Authors:** Annabel Z. Wang, Michael L. Barnett, Jessica L. Cohen

**Affiliations:** 1 Harvard Medical School, Harvard University, Cambridge, Massachusetts; 2 Department of Health Policy and Management, Harvard T.H. Chan School of Public Health, Boston, Massachusetts; 3 Department of Medicine, Brigham and Women’s Hospital, Boston, Massachusetts; 4 Department of Global Health and Population, Harvard T.H. Chan School of Public Health, Boston, Massachusetts

## Abstract

**Importance:**

Although screenings for breast and colorectal cancer are widely recommended, patient screening rates vary greatly and remain below public health targets, and primary care physicians’ (PCPs’) counseling and referrals play critical roles in patients’ use of cancer screenings. Recent adverse events may influence PCPs’ decision-making, but it remains unknown whether cancer screening rates of PCPs’ patients change after PCPs are exposed to new cancer diagnoses.

**Objective:**

To investigate whether PCPs’ exposures to patients with new diagnoses of breast or colorectal cancer were associated with changes in screening rates for other patients subsequently visiting the affected PCPs.

**Design, Setting, and Participants:**

This cohort study used stacked difference-in-differences analyses of all-payer claims data for New Hampshire and Maine in 2009 to 2015. Participants were PCPs caring for patients. Data analysis was performed from June 2020 to May 2022.

**Exposures:**

New diagnosis of a PCP’s patient with breast cancer or colorectal cancer.

**Main Outcomes and Measures:**

Patients’ breast and colorectal cancer screening rates within 1 year of a PCP visit.

**Results:**

The sample included 3158 PCPs (1819 male PCPs [57.6%]) caring for 1 920 189 patients (1 073 408 female patients [55.9%]; mean [SD] age, 41.0 [21.9] years) aged 18 to 64 years. During the study period, 898 PCPs had a patient with a new diagnosis of breast cancer and 370 PCPs had a patient with a new diagnosis of colorectal cancer. In the preexposure period, 68 837 female patients (37.3% of those visiting a PCP) underwent breast cancer screening within 1 year of the visit, and 13 137 patients (10.1% of those visiting a PCP) underwent colorectal cancer screening within 1 year of the visit. For both cancer types, after exposure to a new cancer diagnosis, PCPs’ cancer screening rates displayed a rapid, sustained increase. Breast cancer screening rates increased by 4.5 percentage points (95% CI, 3.0-6.1 percentage points; *P* < .001). Colorectal cancer screening rates increased by 1.3 percentage points (95% CI, 0.3-2.2 percentage points; *P* = .01). Observed breast cancer screening increases were higher for male PCPs than for female PCPs (3.1 percentage points; 95% CI, 0.4-5.8 percentage points; *P* = .03).

**Conclusions and Relevance:**

This study found significant, sustained increases in cancer screening rates for patients visiting PCPs recently exposed to new breast and colorectal cancer diagnoses. These findings suggest that PCPs may update practice patterns on the basis of recent patient diagnoses. Future work should assess whether salient cues to PCPs about patient diagnoses when clinically appropriate can improve screening practices.

## Key Points

**Question:**

Is primary care physician (PCP) exposure to a patient with a new breast or colorectal cancer diagnosis associated with increases in cancer screenings for other patients who subsequently visit the affected PCP?

**Findings:**

In this cohort study of 3158 PCPs caring for 1 920 189 patients, using stacked difference-in-differences analyses, there were rapid and sustained increases in cancer screening rates for patients visiting PCPs who were recently exposed to new cancer diagnoses, for both breast cancer and colorectal cancer.

**Meaning:**

These findings suggest that PCPs’ exposures to new diagnoses of cancer are associated with significant, sustained increases in cancer screening rates for other patients subsequently visiting the exposed PCPs.

## Teaser

This cohort study examines whether primary care physicians’ exposures to patients with new diagnoses of breast or colorectal cancer were associated with changes in screening rates for other patients subsequently visiting the affected physicians.

## Introduction

Despite the availability of effective preventive screenings for breast and colorectal cancers, an estimated 29% of breast cancers and 53% of colorectal cancers are still diagnosed at a late stage annually.^1,2^ Regular preventive screenings could contribute to reductions in mortality and morbidity through earlier diagnoses of these cancers—the second and third most common causes of cancer deaths in the US—while treatment options and curative potential are better.^3,4,5,6,7^ Periodic screenings for breast and colorectal cancers are endorsed by the US Preventive Services Task Force, the American Cancer Society, and numerous other professional organizations.^7,8,9,10,11,12,13,14,15^ However, patient screening rates vary widely and remain below public health targets.^16,17^

Primary care physicians’ (PCPs’) counseling and referrals play a major role in their patients’ use of cancer screenings.^16,18,19,20,21,22^ Clinical guidelines regarding when physicians should refer patients to screening consider evidence-based factors, such as patient age, gender, family history, and environmental exposures.^23,24^ In practice, however, physicians’ decisions to counsel and refer patients on cancer screening may also be informed by inaccurate information or beliefs of cancer risk and by nonclinical heuristics, including situational factors such as appointment time and cognitive load.^16,25,26,27^

One potential factor associated with a PCP’s counseling and referral for cancer screening is the salient event of a diagnosis of new cancer in their patient panel. Evidence from psychology and behavioral economics suggests that salient adverse events can influence provider decision-making.^28,29,30,31^ For example, a 2018 randomized trial^32^ of letters notifying physicians of opioid overdoses in patients to whom they prescribed opioids demonstrated a sharp decrease in prescribing. Additional studies^33,34,35,36,37^ on adverse clinical events, such as gastrointestinal bleeding, pulmonary embolism diagnoses, and unexpected newborn deaths, have found associations between such events and downstream changes in physicians’ clinical decisions. It is plausible that a new cancer diagnosis could increase cancer screening rates through similar behavioral mechanisms. The new diagnosis could act as a salient reminder for PCPs to counsel on preventive screenings, increase PCPs’ estimated likelihood of cancer because of the ease of recalling a recent case (ie, availability bias), or increase physicians’ sense of urgency to recommend screenings more strongly.^28,29,30,31^ In situations in which screenings may be helpful for patients, salient cues such as recent cancer diagnoses may serve as important reminders or motivation for physicians to recommend screenings. However, little is known about whether and how physicians modify their screening recommendations following a new cancer diagnosis among their patients. In this study, we investigated whether PCPs’ exposures to patients with new diagnoses of breast or colorectal cancer were associated with subsequent changes in breast and colorectal cancer screenings for other patients who visited the affected PCPs.

## Methods

### Data and Study Population

This cohort study used commercial insurer and Medicaid claims from the 2009 to 2015 New Hampshire and Maine All-Payer Claims Databases.^38,39^ The institutional review board at the Harvard School of Public Health deemed this study exempt because of the use of secondary deidentified data, in accordance with 45 CFR §46. This study followed the Strengthening the Reporting of Observational Studies in Epidemiology (STROBE) reporting guideline.^40^

The study population included primary care physicians (PCPs) of all patients aged 18 to 64 years for the entire study period (adults younger than the typical Medicare eligibility age).^41,42^ PCPs were included if they held MD or DO degrees, had clinic locations in New Hampshire or Maine, and practiced in internal medicine, family medicine, general practice, or geriatrics (eTable 1 in the Supplement). Patients were attributed to PCPs using the annual plurality of evaluation and management office visits, with ties broken randomly (eTable 1 in the Supplement).^43,44,45^

### Study Exposure

In separate analyses for breast cancer and colorectal cancer, we included the set of all patients attributed to PCPs to identify new cancer diagnoses during the study period. The exposures of interest were new diagnoses of breast or colorectal cancer in a PCP’s patient panel. We identified new diagnoses with *Current Procedural Terminology* (*CPT*) and *International Classification of Disease, Ninth Revision* (*ICD-9*) codes, using procedures adapted from validated claims algorithms (eTable 2 in the Supplement).^46,47,48^ A new diagnosis required the occurrence of both cancer-relevant *ICD-9* and *CPT* codes, with no occurrence of these codes in the previous year (eAppendix in the Supplement). To exclude outlier physicians with few attributed patients, physicians in the lowest fifth percentile of patient panel size were excluded (eAppendix in the Supplement). Consistent with previous literature, we used the first new cancer diagnosis in the study period as the exposure of interest for PCPs with multiple new cancer diagnoses.^33,34,35,36,37^

### Outcome Measures

The study had 2 primary outcomes defined using patient visits and aggregated to the PCP-month level, 1 for breast cancer screening and 1 for colorectal cancer screening. Screening tests were identified by *CPT* and *ICD-9* codes (eTable 3 in the Supplement). For breast cancer, we constructed a binary variable equal to 1 if a female patient underwent mammography within 1 year of visiting her PCP. For colorectal cancer, we constructed a binary variable equal to 1 if a patient underwent colonoscopy, sigmoidoscopy, fecal occult blood testing, fecal immunochemical testing, and/or multitarget stool DNA testing within 1 year of visiting their PCP. We attributed all patient visits to PCPs, aggregated to monthly visit counts per PCP. For each patient visit, we determined whether that patient received the screening test of interest within the following 12 months. This procedure yielded a per-month, per-PCP fraction of patient visits to a PCP resulting in screening completion within the next year (screening rate). Monthly screening rates were further aggregated to the quarterly level for model estimates and plots. We included a broad age window for patients (aged 18-64 years) because screening may be warranted for younger patients with relevant family histories or risk factors; such clinical factors cannot be observed in these data to know whether specific screenings were appropriate.

### Study Covariates

Physician gender and clinical specialties were obtained from the All-Payer Claims Database provider files and cross-checked in the National Provider and Plan Enumeration System data set and Doctors and Clinicians National File (Physician Compare, Centers for Medicare & Medicaid Services) using National Provider Identifiers.^49,50^ Physicians’ years of experience were estimated using medical school graduation years. Patient age (via date of birth), gender, and insurance status were obtained from All-Payer Claims Database beneficiary files.

### Statistical Analysis

Data analysis was performed from June 2020 to May 2022. We used a stacked difference-in-differences (DD) study design,^51,52^ with PCP-quarter as the unit of analysis (eFigure in the Supplement). We compared changes in screening rates for PCPs following exposure to a new cancer diagnosis (currently exposed PCPs) to changes in screening rates for PCPs who were not yet exposed to a patient with a new cancer diagnosis but would be later in the study period (future-exposed PCPs). Stacked DD methods use future-exposed PCPs as the comparison group for 2 reasons. First, never-exposed PCPs may differ in critical ways from ever-exposed PCPs and, thus, may not be a valid comparison group (eTable 4 in the Supplement). Second, previously exposed PCPs may still be experiencing time-varying treatment effects and, thus, may induce bias if included as comparison with currently exposed PCPs. Inference from stacked DD analyses requires that, in the absence of study exposure, differences between outcomes for the treatment (currently exposed) and comparison (future-exposed) PCPs would remain constant over time.^53^

We tested for parallel preexposure trends in outcomes between treatment and comparison groups, using *F* tests of coefficients with the null hypothesis that preexposure coefficients were jointly equal to 0. We analyzed changes in screening rates over 4 quarters before and after a new cancer diagnosis.^33,35,36,37^ Index patients whose new breast or colorectal cancer diagnoses were the study exposures were excluded from study outcomes to avoid a mechanical correlation between exposures and outcomes.

The key variables in the stacked DD model were a set of indicators for each quarter relative to new cancer exposure (ie, −4 to 4 quarters) and an indicator for whether the PCP was in the currently exposed group (vs future-exposed group). To adjust for potential confounding by time-invariant PCP characteristics (eg, gender, age, and race), the model included indicator variables for each PCP. The model included indicator variables for each year and calendar month to adjust for time-varying and seasonal factors affecting PCPs’ screening recommendations. We used robust SEs clustered at the PCP level. Analyses were conducted using Stata statistical software version 17 (StataCorp, LLC). Two-sided hypothesis tests used a significance threshold of *P* < .05.

In additional subgroup analyses, we assessed whether model estimates varied by physician characteristics. PCP characteristics included gender (male vs female), years of clinical experience (divided at median, >18 years vs ≤18 years in practice), and panel composition by patient insurance type (proportion of patient panel enrolled in Medicaid vs commercial insurance). We tested for differences in means between subgroups, using subgroup-specific variables interacted with the main effect term in separate analyses. We also conducted colorectal cancer analyses stratified by screening modality.

As a robustness check, we conducted falsification tests for each analysis, using breast cancer diagnoses as index events for colorectal cancer screening rates in stacked DD models and vice versa. Screening rates would not be expected to change for 1 cancer type following the diagnosis of a different cancer type and, if we saw such an association, it would suggest potential confounding. We also assessed for sensitivity of findings to varying specifications, including sample and exposure criteria (separate analyses, excluding PCPs with more than 2 total exposures; exposures followed by a subsequent exposure within 1 year; carcinoma in situ diagnosis codes; and patients younger than 35, 40, and 50 years [separate analyses]), covariates used (calendar month vs calendar quarter indicators, and month vs year indicators), and number of quarters included before and after index events.

## Results

The study sample included 3158 PCPs (1819 male PCPs [57.6%]) caring for 1 920 189 patients (1 073 408 female patients [55.9%]; mean [SD] age, 41.0 [21.9] years; age range, 18-64 years). From 2009 to 2015, 898 PCPs had a patient with a new diagnosis of breast cancer, and 370 PCPs had a patient with a new diagnosis of colorectal cancer (Table 1). Currently exposed and future-exposed PCPs had similar characteristics at the start of the study period (eTable 4 in the Supplement).

**Table 1.** Characteristics of PCPs in Breast Cancer and Colorectal Cancer Analyses^a^

Characteristic	PCPs exposed to new cancer diagnosis, No. (%)	
	Breast cancer analysis (n = 898)	Colorectal cancer analysis (n = 370)
Demographic characteristics		
Gender		
Female	348 (38.8)	131 (35.4)
Male	550 (61.2)	239 (64.6)
Years in practice, mean (SD)	19.3 (9.1)	19.8 (9.2)
Clinical specialty		
Family practice	602 (67.1)	257 (69.4)
Internal medicine	286 (31.8)	109 (29.6)
Other medical specialist	10 (1.1)	4 (1.0)
Practice location		
Urban (metropolitan)	513 (57.1)	217 (58.6)
Large rural (micropolitan)	180 (20.1)	78 (21.0)
Small rural	118 (13.1)	39 (10.5)
Isolated rural	87 (9.7)	36 (9.9)
PCP patient panel characteristics, mean (SD)		
Non-Medicare patients in panel, No.	330.6 (217.5)	298.2 (230.3)
Female patients in panel, %	58.3 (15.0)	56.5 (14.3)
Patients enrolled in Medicaid in panel, %	14.6 (13.6)	14.9 (13.1)
Monthly patient visits	68.9 (44.4)	63.7 (38.1)
Female only	42.5 (27.8)	NA
Monthly screenings among patients who visited PCP	NA	4.5 (4.8)
Female only	9.4 (6.6)	NA

Abbreviations: NA, not applicable; PCP, primary care physician.

^a^ Table uses data from quarter-to-event −4 through −1 (preexposure period). Patient panel sizes include only non-Medicare patients. For the breast cancer analysis, monthly patient visits and screenings are also shown for female patients separately, as exposures (breast cancer) and outcomes (mammography screening rates) were assessed for female patients.

### Preexposure

**Figure 1.**
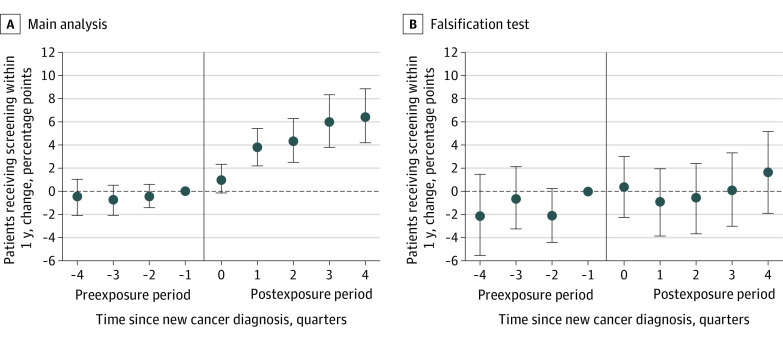
Breast Cancer Screening Rates Among Primary Care Physicians’ (PCPs’) Other Female Patients Following PCP Exposure to a New Breast Cancer Diagnosis Breast cancer screenings included mammography. The quarter of PCP exposure to a new cancer diagnosis is denoted by relative quarter 0 (0 quarters since new cancer diagnosis). Each relative quarter data point represents the difference between treatment and comparison PCPs in screening rates in that quarter relative to the quarter before exposure (difference-in-differences in outcome, relative to quarter −1). The outcome is defined as the proportion of patients visiting a PCP in a given quarter who undergo breast cancer screening within 1 year of the PCP visit. Index patients whose diagnoses were the PCPs’ exposures were excluded from analyses. Estimates are expressed in percentage points with 95% CIs (error bars), which were estimated using robust SEs clustered at the PCP level. Panel A shows the main breast cancer analysis (*P* = .44 for joint significance test of preexposure coefficients in main analysis; preexposure screening rate, 37.3%). Panel B shows the falsification test for breast cancer analysis, where breast cancer screening outcomes are plotted, but exposures were colorectal cancer diagnoses (*P* = .27 for joint significance test of preexposure coefficients in falsification test; preexposure screening rate, 40.5%).

**Figure 2.**
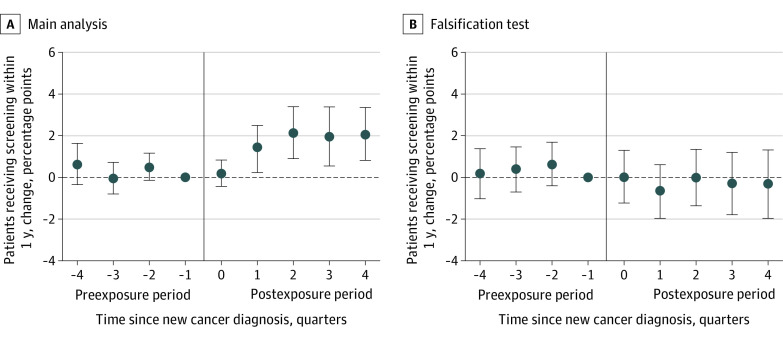
Colorectal Cancer Screening Rates Among Primary Care Physicians’ (PCPs’) Other Patients Following PCP Exposure to a New Colorectal Cancer Diagnosis Colorectal cancer screenings included colonoscopy, sigmoidoscopy, fecal immunochemical testing, fecal occult blood testing, and/or multitarget stool DNA tests. The quarter of PCPs’ exposures to a new cancer diagnosis is denoted by relative quarter 0 (0 quarters since new cancer diagnosis). Each relative quarter data point represents the difference between treatment and comparison PCPs in screening rates in that quarter relative to the quarter before exposure (difference-in-differences in outcome, relative to quarter −1). The outcome is defined as the proportion of patients visiting a PCP in a given quarter who receive colorectal cancer screening within one year of the PCP visit. Index patients whose diagnoses were the PCP exposures were excluded from analyses. Estimates are expressed in percentage points with 95% CIs (error bars), which were estimated using robust SEs clustered at the PCP level. Panel A shows the main colorectal cancer analysis (*P* = .13 for joint significance test of preexposure coefficients in main analysis; preexposure screening rate, 10.1%). Panel B shows the falsification test for colorectal cancer analysis, where colorectal cancer screening outcomes are plotted, but exposures were breast cancer diagnoses (*P* = .58 for joint significance test of preexposure coefficients in falsification test; preexposure screening rate, 12.4%).

Statistical tests failed to reject the null hypothesis of parallel trends—that all preexposure coefficients were jointly equal to 0—in screening rates between treatment and comparison PCPs. Relevant data are shown in Figure 1 and Figure 2.

### Breast Cancer Analysis

Following exposure to a new breast cancer diagnosis, breast cancer screening rates among PCPs’ patient panels displayed a rapid and sustained increase (Figure 1 and Table 2). By the fourth quarter after exposure, breast cancer screening rates were 6.5 percentage points higher than the quarter before exposure (95% CI, 4.2 to 8.9 percentage points; *P* < .001). On the basis of a preexposure screening rate of 37.3% (68 837 patients) among female patients younger than the Medicare eligibility age, this change represents a relative 17.4% increase in the fraction of patients visiting an exposed PCP who underwent a mammogram within 1 year. The overall screening increase observed during the postexposure period was 4.5 percentage points (95% CI, 3.0 to 6.1 percentage points; *P* < .001), a 12.1% increase from baseline. Falsification tests did not find significant differences in breast cancer screening rates following colorectal cancer diagnoses (absolute change, 0.8 percentage points; 95% CI, −1.5 to 3.2 percentage points; *P* = .49) (eTable 5 in the Supplement).

**Table 2.** Postexposure Change in Proportion of Patients Who Undergo Cancer Screening Within Next Year

Time period relative to exposure	Breast cancer analysis			Colorectal cancer analysis		
	Absolute change, percentage points (95% CI)	Relative change, % (95% CI)^a^	*P* value	Absolute change, percentage points (95% CI)	Relative change, % (95% CI)^b^	*P* value
Preexposure period (quarters −4 to −1)	0 [Reference]	0 [Reference]	NA	0 [Reference]	0 [Reference]	NA
Overall postexposure change	4.5 (3.0-6.1)	12.1 (8.0-16.4)	<.001	1.3 (0.3-2.2)	12.9 (3.0-21.8)	.01
Quarter 1	3.8 (2.2-5.4)	10.2 (5.9-14.5)	<.001	1.4 (0.3-2.5)	13.9 (3.0-24.8)	.02
Quarter 2	4.4 (2.5-6.3)	11.8 (6.7-16.9)	<.001	2.2 (0.9-3.4)	21.8 (8.9-33.7)	.001
Quarter 3	6.1 (3.8-8.3)	16.4 (10.2-22.3)	<.001	2.0 (0.5-3.4)	19.8 (5.0-33.7)	.01
Quarter 4	6.5 (4.2-8.9)	17.4 (11.3-23.9)	<.001	2.1 (0.8-3.4)	20.8 (7.9-33.7)	.001

^a^ The breast cancer main analysis preexposure screening rate was 37.3%.

^b^ The colorectal cancer main analysis preexposure screening rate was 10.1%.

### Colorectal Cancer Analysis

After exposure to a new colorectal cancer diagnosis, colorectal cancer screening rates among PCPs’ patient panels also demonstrated a rapid and sustained increase (Figure 2 and Table 2). By the fourth quarter, colorectal cancer screening rates were 2.1 percentage points higher than the quarter before exposure (95% CI, 0.8 to 3.4 percentage points; *P* = .001). This change represents a relative 20.8% increase in the fraction of patients visiting an exposed PCP who underwent a colorectal cancer screening within 1 year of a visit (10.1% preexposure screening rate; 13 137 patients). The overall screening increase observed during the postexposure period was 1.3 percentage points (95% CI, 0.3 to 2.2 percentage points; *P* = .01), a 12.9% increase from baseline. Falsification tests did not find significant differences in colorectal cancer screening rates following breast cancer diagnoses (absolute change, −0.8 percentage points; 95% CI, −1.9 to 0.2 percentage points; *P* = .13) (eTable 5 in the Supplement).

### Subgroup Analyses and Sensitivity Tests

Postexposure screening increases were higher for male PCPs compared with female PCPs in the breast cancer analysis (3.1 percentage points; 95% CI, 0.4 to 5.8 percentage points; *P* = .03) (Table 3), but not in the colorectal cancer analysis (−0.01 percentage point; 95% CI, −2.00 to 2.00 percentage points; *P* = .99). There were no detected differences across subgroups of physician clinical experience (>18 years vs ≤18 years in practice) or patient panel insurance type composition (Table 3). Findings were consistent in sensitivity tests of varying sample size and exposure exclusion criteria, covariates, and analytical window length, although the findings were smaller and less precise for subgroup analyses excluding younger patients (eTables 6-13 in the Supplement).

**Table 3.** Subgroup Analyses, Postexposure Change in Proportion of Patients Who Receive Cancer Screening Within Next Year

Exposure group of interest	Breast cancer analysis		Colorectal cancer analysis	
	Absolute change, percentage points (95% CI)	*P* value	Absolute change, percentage points (95% CI)	*P* value
Preexposure period (quarters −4 to −1)	0 [Reference]	NA	0 [Reference]	NA
Main analyses, all PCPs (overall postexposure change)	4.5 (3.0 to 6.1)	<.001	1.3 (0.3 to 2.2)	.01
Subgroup analyses (difference between subgroups in overall postexposure change)				
Male PCP vs female PCP	3.1 (0.4 to 5.8)	.03	−0.01 (−2.0 to 2.0)	.99
PCP clinical experience, >18 y vs ≤18 y in practice	2.8 (−0.4 to 5.9)	.09	−0.1 (−2.1 to 2.0)	.95
Patient insurance type, proportion of PCPs’ patients enrolled in Medicaid	0.5 (−9.1 to 10.0)	.92	0.4 (−6.1 to 7.0)	.90

Abbreviation: PCP, primary care physician.

## Discussion

This cohort study found that breast and colorectal cancer screening rates increased among patients visiting a PCP soon after a patient in the PCP’s panel received a new diagnosis of cancer. The increases in screening rates were not observed until after PCPs were exposed to a new cancer diagnosis and were sustained for at least a year after exposure. For breast cancer, the magnitude of screening increases was larger for male PCPs than female PCPs. This study highlights one example of how physicians’ experiences—here, exposure to a new cancer diagnosis—may correspond with the rates at which physicians’ other patients are screened for disease, apart from or in addition to baseline consideration of evidence-based risk factors. Furthermore, physician characteristics, such as gender, may affect responses to exposures.

There are various potential reasons for why a PCP’s patients’ cancer screening rates may increase following a new cancer diagnosis. First, PCPs may update their assessment of their patients’ risk of cancer, either for all patients or patients with specific characteristics. This increase in a PCP’s subjective assessment of patients’ risks could lead them to suggest screenings to an increased number of patients and put more effort into counseling patients on the benefits of screening. Additionally, a recent diagnosis may induce an availability bias into PCPs’ decisions on cancer screening, which has been associated in other clinical settings with changes in physicians’ decision-making. Availability bias occurs when recent events are overemphasized in decision-making because of ease of recalling those events.^29^ Even if PCPs intend to recommend cancer screenings, they may forget or be unable to put sufficient effort into counseling on the benefits of screening because of time pressure from high scheduling commitments. A recent cancer diagnosis may bring cancer to top of mind during patient visits, making the importance of counseling on cancer screenings more salient at a key time when mental bandwidth may be low.^30,31^ Previous evidence suggests that PCP referrals for breast and colorectal cancer screening decline over the course of the day, suggesting that cognitive load and fatigue may play important roles in cancer screening rates.^25^ Overall, a patient’s new cancer diagnosis likely corresponds with changes in a physician’s judgment and/or emotion, reflected in modified physician decision-making and effort through a range of mechanisms, many of which are not mutually exclusive.

Our results contribute to the developing literature on whether and how physician behavior changes after adverse events, extending this concept to diagnoses and screenings for breast cancer and colorectal cancer, 2 leading causes of cancer mortality in the US and worldwide.^7^ Breast and colorectal cancer diagnoses are common exposures across many PCPs’ careers, yet to our knowledge, have not been studied as potential adverse event exposures in context of physician decision-making. Prior related studies^34,35,36,37^ have reported changes in physicians’ decisions in areas such as blood thinning medication prescriptions, cesarean deliveries, and pulmonary embolism testing. Another study^33^ investigated colonoscopy-induced gastrointestinal bleeding and subsequent reductions in physicians’ colonoscopy orders. Our observed magnitudes of change lie in a similar range as estimates in these studies and were sustained throughout the study period.^33,34,35,36,37^ Additionally, this study contributes methodologically through its use of all-payer claims data to conduct longitudinal PCP-level analyses of practice pattern changes in relation to patient diagnoses and outcomes. This study is not limited to 1 specific health care system, but rather makes use of commercial and Medicaid claims for all primary care practices across 2 states.

Although PCPs are increasingly held accountable for quality measures that incorporate screening tests, the feedback PCPs receive is often limited to performance in those metrics rather than clinically meaningful and salient cues, such as targeted updates about their patients.^54,55,56,57,58,59,60^ Furthermore, although effective screenings are available for breast and colorectal cancer, such screenings remain underutilized.^16,17^ Understanding the factors associated with PCPs’ varying practice patterns in relation to cancer screening is, thus, of prime importance. This study adds to the evidence that physicians may update their practice patterns according to patients’ diagnoses or outcomes when they learn about them. Cues or nudges based on adverse events may have variable health impacts, depending on whether a screening is warranted; when screenings are beneficial to patients, informing physicians of such events could be helpful. If implemented in clinically appropriate situations, the provision of salient, patient-focused information or cues to physicians could provide useful, targeted, and motivating guidance for PCPs as they care for patients.

### Limitations

This study has limitations that should be addressed. The analysis was an observational study, with specific assumptions for interpreting the findings. The study was limited to 2 states, New Hampshire and Maine. Generalizability to other settings or cancer types is uncertain and should be tested. New cancer diagnoses, attribution of patients to PCPs, and screenings were identified using claims-based algorithms. Although the study adapted algorithms validated in peer-reviewed literature, claims-based methods can introduce imprecision, such as through the inability to confirm whether a PCP directly ordered an observed screening. Additionally, the data did not allow for analyses that determine whether individual screenings were appropriate according to clinical risk factors or distinguish preventive from diagnostic screenings. The study does not reveal the specific mechanism for associations observed.

## Conclusion

This study highlights significant, sustained increases in PCPs’ cancer screening rates following exposures to new cancer diagnoses, in the context of both breast cancer and colorectal cancer, 2 major contributors to cancer morbidity and mortality. Future evidence will be imperative to assess whether methods to provide PCPs with salient information cues about patient diagnoses and outcomes in clinically appropriate situations can improve evidence-based screening practices.

## Notes

Supplement. **eAppendix.** Data Details and Study Design **eFigure.** Illustration of Stacked Difference-in-Differences Study Design **eTable 1.** Identification of Primary Care Physicians and Visits **eTable 2.** Identification of New Cancer Diagnoses **eTable 3.** Identification of Preventive Cancer Screenings **eTable 4.** Characteristics of Currently-Exposed, Future-Exposed, and Never-Exposed Primary Care Physicians **eTable 5.** Falsification Test Results for Breast Cancer and Colorectal Cancer Analyses **eTable 6.** Robustness Tests: Results From Alternative Regression Specifications **eTable 7.** Sensitivity Analyses: Breast Cancer Analysis With Sequential Patient Age Restrictions **eTable 8.** Sensitivity Analyses: Colorectal Cancer Analysis With Sequential Patient Age Restrictions **eTable 9.** Sensitivity Analysis: No Carcinoma In Situ Diagnoses **eTable 10.** Sensitivity Analysis: No Exposures Followed by a Subsequent Exposure Within the Same Year **eTable 11.** Sensitivity Analysis: PCPs With Two or Fewer Total Exposures **eTable 12.** Sensitivity Analysis: 8-Quarter Analytical Window **eTable 13.** Additional Analysis: Colorectal Cancer Analysis, Stratified by Categories of Cancer Screening Modality
eReferences
