# Supplementary material for: Changes in Cancer Screening Rates Following a New Cancer Diagnosis in a Primary Care Patient Panel
Source: JAMA Netw Open. 2022 Jul 15;5(7):e2222131. doi: 10.1001/jamanetworkopen.2022.22131 (PMC9287757; doi:10.1001/jamanetworkopen.2022.22131)
Supplement: Supplement. — eAppendix. Data Details and Study Design eFigure. Illustration of Stacked Difference-in-Differences Study Design eTable 1. Identification of Primary Care Physicians and Visits eTable 2. Identification of New Cancer Diagnoses eTable 3. Identification of Preventive Cancer Screenings eTable 4. Characteristics of Currently-Exposed, Future-Exposed, and Never-Exposed Primary Care Physicians eTable 5. Falsification Test Results for Breast Cancer and Colorectal Cancer Analyses eTable 6. Robustness Tests: Results From Alternative Regression Specifications eTable 7. Sensitivity Analyses: Breast Cancer Analysis With Sequential Patient Age Restrictions eTable 8. Sensitivity Analyses: Colorectal Cancer Analysis With Sequential Patient Age Restrictions eTable 9. Sensitivity Analysis: No Carcinoma In Situ Diagnoses eTable 10. Sensitivity Analysis: No Exposures Followed by a Subsequent Exposure Within the Same Year eTable 11. Sensitivity Analysis: PCPs With Two or Fewer Total Exposures eTable 12. Sensitivity Analysis: 8-Quarter Analytical Window eTable 13. Additional Analysis: Colorectal Cancer Analysis, Stratified by Categories of Cancer Screening Modality eReferences [file jamanetwopen-e2222131-s001.pdf]

Supplemental Online Content

Wang AZ, Barnett ML, Cohen JL. Changes in cancer screening rates following a new cancer diagnosis in a primary care patient panel. *JAMA Netw Open*. 2022;5(7):e2222131. doi:10.1001/jamanetworkopen.2022.22131

eAppendix. Data Details and Study Design
eFigure. Illustration of Stacked Difference-in-Differences Study Design
eTable 1. Identification of Primary Care Physicians and Visits
eTable 2. Identification of New Cancer Diagnoses
eTable 3. Identification of Preventive Cancer Screenings
eTable 4. Characteristics of Currently-Exposed, Future-Exposed, and Never-Exposed Primary Care Physicians
eTable 5. Falsification Test Results for Breast Cancer and Colorectal Cancer Analyses
eTable 6. Robustness Tests: Results From Alternative Regression Specifications
eTable 7. Sensitivity Analyses: Breast Cancer Analysis With Sequential Patient Age Restrictions
eTable 8. Sensitivity Analyses: Colorectal Cancer Analysis With Sequential Patient Age Restrictions
eTable 9. Sensitivity Analysis: No Carcinoma In Situ Diagnoses
eTable 10. Sensitivity Analysis: No Exposures Followed by a Subsequent Exposure Within the Same Year
eTable 11. Sensitivity Analysis: PCPs With Two or Fewer Total Exposures
eTable 12. Sensitivity Analysis: 8-Quarter Analytical Window
eTable 13. Additional Analysis: Colorectal Cancer Analysis, Stratified by Categories of Cancer Screening Modality
eReferences

This supplemental material has been provided by the authors to give readers additional information about their work.

© 2022 Wang AZ et al. *JAMA Network Open.*

eAppendix. Data Details and Study Design

A. Identification of Primary Care Physicians and Visits

Primary care physicians (PCPs) were identified using taxonomy codes indicated by the Centers for Medicare and Medicaid Services (CMS) to represent primary care specialties (eTable 1).

B. Identification of New Diagnoses of Breast Cancer and Colorectal Cancer

New diagnoses of breast cancer and colorectal cancer were identified using International Classification of Disease (ICD) and Current Procedural Terminology (CPT)/Healthcare Common Procedure Coding System (HCPCS) codes (eTable 2). Beneficiaries were identified who had at least one qualifying cancer diagnosis code (ICD) and at least one procedure code (CPT/HCPCS) in their claims, where the earliest claim with a code of interest was not preceded by any diagnosis or procedure codes of interest in the prior year. Month of new cancer diagnosis ("diagnosis month") was marked as the earliest month where an applicable code was present.

The month of interest in this study was the month of a given PCP's first incidence of exposure to a new cancer diagnosis among his or her patients. To ensure that exposures were recorded as early as applicable, the exposure month was calculated as either a) the earliest diagnosis month or b) the month of a relevant preventive cancer screening if the screening occurred in the two months immediately prior to the earliest diagnosis month (i.e., the month identified in (a)). Qualifying ICD codes, CPT/HCPCS codes, and algorithms for identifying new diagnoses were adapted from previously validated claims-based algorithms.[1–3] Months of exposure were converted to quarters of exposure for the final aggregated analysis.

C. Preventive Cancer Screenings

Preventive screenings for breast cancer and colorectal cancer were identified using ICD and CPT/HCPCS codes (screening or diagnostic; eTable 3).

D. Stacked Difference-in-Differences Sample and Study Design[4,5]

In the stacked difference-in-differences analytical structure, the study sample is restricted to PCPs who are exposed at some point in the data period (i.e., no "never-exposed" PCPs). The exposure of interest is the first incidence of a new diagnosis of breast or colorectal cancer in a PCP's patient. In each quarter, a "currently-exposed" PCP represents a PCP who is exposed to a new cancer diagnosis in that quarter. A "future-exposed" PCPs represents a PCP who is not exposed to a new cancer diagnosis in that quarter, but who will be exposed to a new cancer diagnosis later in the study period (greater than four quarters in the future). In the stacked difference-in-differences model, the pre-exposure period for future-exposed PCPs is used as a comparison for currently-exposed PCPs.

© 2022 Wang AZ et al. *JAMA Network Open.*

To construct the analytical dataset, for a given quarter, we denote PCPs who experience an exposure in the current quarter as treatment PCPs, and PCPs who experience an exposure in the future (more than 4 quarters in the future) as comparison PCPs (eFigure). The sample was constructed by repeating this procedure for each of the 84 quarters over the entire data period, then appending the resulting quarter-specific datasets into one dataset. Note that PCPs can only be in the treatment group in one quarter (PCP's quarter of earliest exposure to a new cancer diagnosis) but can be in the comparison group in multiple quarters. Thus, the sum of currently-exposed PCPs and future-exposed PCPs (eTable 4) is greater than the number of individual PCPs in the study sample.

To exclude outlier physicians with few attributed patients, physicians in the lowest fifth percentile of patient panel size were excluded. In the breast cancer analysis, the mean annual patient panel size for PCPs in the lowest fifth percentile of panel size was 38.7 patients (SD = 4.7). In the colorectal cancer analysis, the mean annual patient panel size for PCPs in the lowest fifth percentile of panel size was 54.4 patients (SD = 10.8).

The outcome measure was calculated at the PCP-quarter level, representing the proportion of patients who visited a PCP in a given quarter who received a relevant cancer screening within a year of that PCP visit. The stacked difference-in-differences model calculated the difference between treatment and comparison PCPs in screening rates (outcome measure) before vs. after exposure to a new cancer diagnosis. The quarter of exposure (relative quarter 0) was included in the exposure period because it is possible for some effect to already occur during the quarter of exposure. The model included a vector of calendar month indicator variables (calendar month fixed effects), year indicator variables (year fixed effects), and PCP indicator variables (PCP fixed effects).

Additionally, we estimated a "relative quarter" version of the main model, where each relative quarter coefficient represented the difference between treatment and comparison PCPs in screening rates in that quarter relative to the quarter prior to exposure to a new cancer diagnosis (relative quarter -1).

Subgroup analyses, which estimated the relative effect sizes of subgroups of PCP characteristics, were estimated through the coefficient of an interaction term between the subgroup characteristic of interest (as an indicator variable) and the main model effect coefficient.

All models were estimated using robust standard errors clustered at the PCP level.

F. Tests of Pre-Exposure Parallel Trends

An identifying assumption for difference-in-differences requires that in the absence of study exposure, differences in outcomes for the treatment and comparison PCP groups would remain constant over time. Though this assumption cannot be explicitly tested, a test of pre-exposure parallel trends can aid in examining the likelihood that it holds. We tested for differential changes between treatment and comparison groups prior to exposure, using F-tests of pre-exposure coefficients, where a null finding provides support for parallel trends (by signifying there is no evidence to reject the null of parallel trends). Null findings demonstrate that the

© 2022 Wang AZ et al. *JAMA Network Open.*

differences in outcomes for these groups were not showing significant signs of deviating from each other for a considerable period before exposure (-Q4 through -Q1). Specifically, the test assessed whether the difference-in-difference between relative quarters -Q4, -Q3, -Q2, and -Q1 (pre-exposure, i.e., before Q0) was different for PCPs who would be treated at Q0 (treatment, currently-exposed PCPs) vs. PCPs who would be treated later than Q0 (comparison, future-exposed PCPs). As noted previously, Q0 was included in the exposure period because it is possible for some effect to already occur during the quarter of exposure.

F. Falsification Tests for Breast Cancer and Colorectal Cancer Analyses

In the breast cancer falsification test, colorectal cancer diagnoses were used as index events for breast cancer screening rates in the stacked DD model as specified in the main breast cancer analysis. In the colorectal cancer falsification test, breast cancer diagnoses were used as index events for colorectal cancer screening rates in the stacked DD model as specified in the main colorectal cancer analysis. Results are included in eTable 5.

G. Sensitivity Analyses and Additional Analyses

We conducted additional analyses using varying specifications, including sample and exposure criteria, as detailed below.

Analyses:

1. Using calendar quarter indicators instead of calendar month indicators (eTable 6).
2. Using quarter indicators instead of year indicators (eTable 6).
3. Using month indicators instead of quarter indicators (eTable 6).
4. Including all PCPs rather than excluding PCPs in the lowest fifth percentile of panel size (eTable 6).
5. Using varying analytical windows of analysis (+/- 3 quarters, +/- 5 quarters, +/- 8 quarters (eTable 6; more detailed results for +/- 8 quarters in eTable 12).
6. Restricting outcome measure calculations to sequentially older patient age groups (35-64 years of age, 40-64 years of age, 50-64 years of age [eTables 7 and 8]).
7. Excluding carcinoma in situ diagnosis codes (eTable 9).
8. Excluding exposures to new cancer diagnoses if they were followed by a subsequent exposure within the same year (eTable 10).
9. Excluding PCPs with more than two total exposures over the data period (eTable 11).
10. Stratifying the colorectal cancer analysis by cancer screening modalities (colonoscopy, FOBT/FIT, and sigmoidoscopy in separate analyses [eTable 13]).

© 2022 Wang AZ et al. *JAMA Network Open.*

eFigure. Illustration of Stacked Difference-in-Differences Study Design [a,b]

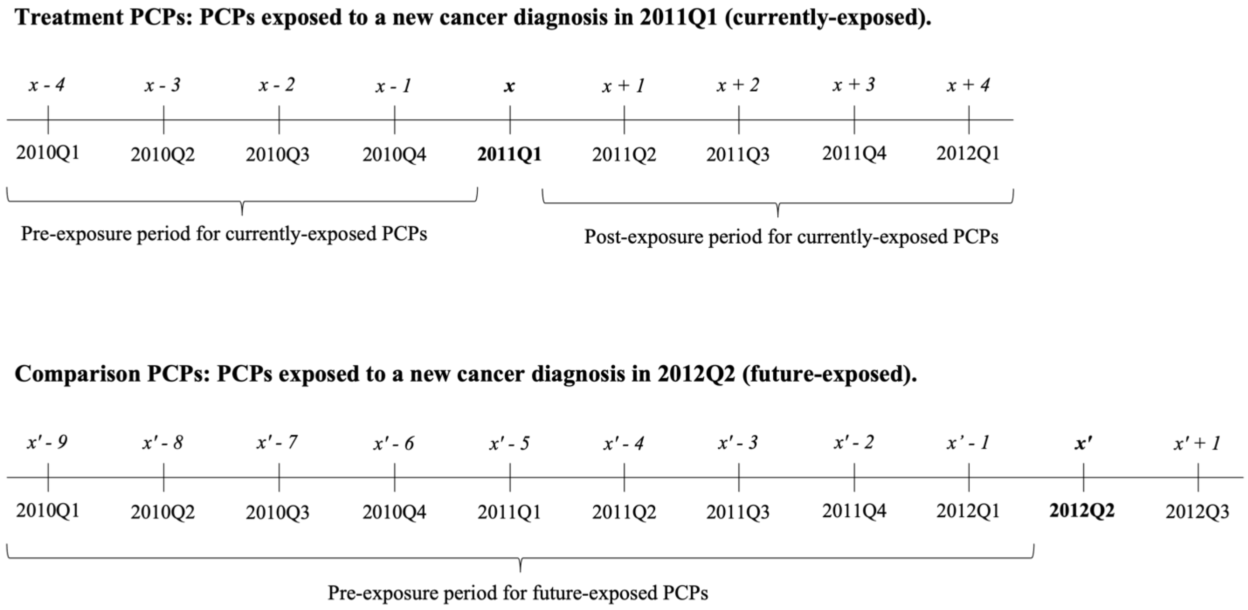

[a] In the stacked difference-in-differences model, the pre-exposure period for future-exposed PCPs is used as a comparison for currently-exposed PCPs. The outcome measure is calculated in each quarter above, as the proportion of patients who visit a PCP in that quarter who receive a relevant cancer screening within a year of the PCP visit. Relative quarter 0 (quarters *x* and *x'*) is included in the exposure period because it is possible for some effect to already occur during the quarter of exposure.

[b] Adaptation of illustration created by Han et al. (JAMA Network Open, 2020).[6]

© 2022 Wang AZ et al. *JAMA Network Open.*

eTable 1. Identification of Primary Care Physicians and Visits

Taxonomy Code	Specialty
208D00000X	(01-General Practice)
207Q00000X	(08-Family Medicine)
207R00000X	(11-Internal Medicine)
207QG0300X	(38-Family Medicine-Geriatric)
207RG0300X	(38-Internal Medicine-Geriatric)
Evaluation and management (E&M) office visits included the subset of claims with at least one CPT/HCPCS code of: 99201-99205, 99211-99215, 99241-99245, G0402, G0438, and G0439.	

© 2022 Wang AZ et al. *JAMA Network Open.*

eTable 2. Identification of New Cancer Diagnoses

**Breast Cancer Analysis**		
**Diagnosis or Procedure**	**ICD Codes**	**CPT/HCPCS Codes**
Breast cancer	174.0-174.9	
Carcinoma in situ	233.0	
Biopsy		19000, 19001, 19100, 19101, 19110, 19112
Lumpectomy		19120, 19125, 19126
Partial mastectomy		19160, 19162
Lymph node dissection		38740, 38745, 38525
Mastectomy		19180-19255, 19301-19307
**Colorectal Cancer Analysis**		
**Diagnosis or Procedure**	**ICD Codes**	**CPT/HCPCS Codes**
Colorectal cancer	153.0-153.9, 154.0-154.8	
Carcinoma in situ	230.3-230.6	
Partial resection of colon		44140, 44141, 44143, 44144-44147
Removal of colon		44150, 44151, 44155-44158, 44160
Removal of rectum		45110-45114, 45116, 45119-45121, 45123

© 2022 Wang AZ et al. *JAMA Network Open.*

eTable 3. Identification of Preventive Cancer Screenings

**Breast Cancer Analysis**	
**Screening**	**CPT/HCPCS Codes**
Mammography	76082, 76083, 76085, 76086, 76088, 76090-76092, 77051, 77052, 77055-77057, G0202-G0207
**Colorectal Cancer Analysis**	
**Screening**	**CPT/HCPCS Codes**
Colonoscopy	44388, 44389, 44392-44394, 44397, 45378-45389-45393, 45398, G0105, G0106, G0120, G0122
Sigmoidoscopy	G0104, G6022, G6023, 45303, 45305, 45307-45309, 45315, 45317, 45320, 45321, 45327, 45330-45334, 45337-45342, 45345
Fecal immunochemical test (FIT) for blood	82274, G0328
Guaiac-based fecal occult blood test (gFOBT)	82270, 82272, G0107
Multitarget stool DNA testing (i.e., sDNA-FIT, MT-sDNA, FIT-DNA, or Cologuard)	S3890

© 2022 Wang AZ et al. *JAMA Network Open.*

eTable 4. Characteristics of Currently-Exposed, Future-Exposed, and Never-Exposed Primary Care Physicians [a]

	Breast Cancer Analysis				Colorectal Cancer Analysis			
	PCPs in Analysis				PCPs in Analysis			
	All PCPs in Analysis N=898[b]	Currently-Exposed PCPs N=567	Future-Exposed PCPs N=398	Never-Exposed PCPs N=2260	All PCPs in Analysis N=370[b]	Currently-Exposed PCPs N=277	Future-Exposed PCPs N=183	Never-Exposed PCPs N=2788
**PCP Characteristics**								
Female PCPs, %	38.8 (N=348)	36.5 (N=207)	39.9 (N=159)	43.7 (N=988)	35.4 (N=131)	35.7 (N=99)	37.0 (N=68)	43.7 (N=1218)
Years in practice, mean (SD)	19.3 (9.1)	19.3 (9.3)	19.6 (8.6)	15.4 (9.5)	19.8 (9.2)	20.0 (9.3)	18.8 (8.6)	16.4 (9.5)
**Clinical specialty**								
Family practice, %	67.1 (N=602)	66.4 (N=376)	64.7 (N=257)	55.9 (N=1263)	69.4 (N=257)	69.4 (N=192)	68.9 (N=126)	59.6 (N=1663)
Internal medicine, %	31.8 (N=286)	32.5 (N=185)	34.3 (N=137)	43.9 (N=992)	29.6 (N=109)	29.8 (N=83)	29.9 (N=55)	39.9 (N=1113)
Other medical specialist, %	1.1 (N=10)	1.1 (N=6)	1.0 (N=4)	0.2 (N=5)	1.0 (N=4)	0.8 (N=2)	1.2 (N=2)	0.4 (N=12)
**Practice Location**								
Urban (metropolitan), %	57.1 (N=513)	52.2 (N=296)	56.0 (N=223)	51.6 (N=1166)	58.6 (N=217)	59.0 (N=163)	57.9 (N=106)	51.8 (N=1445)
Large rural (micropolitan), %	20.1 (N=180)	23.6 (N=134)	21.4 (N=85)	19.7 (N=445)	21.0 (N=78)	21.5 (N=60)	20.5 (N=38)	20.1 (N=560)
Small rural, %	13.1 (N=118)	12.9 (N=73)	13.5 (N=54)	15.6 (N=353)	10.5 (N=39)	9.6 (N=27)	12.8 (N=23)	15.5 (N=432)
Isolated rural, %	9.7 (N=87)	11.3 (N=64)	9.1 (N=36)	13.1 (N=297)	9.9 (N=36)	9.9 (N=27)	8.8 (N=16)	12.6 (N=351)
**Patient Panel Characteristics [Non-Medicare]**								
Number of patients in panel [non-Medicare], mean (SD)	330.6 (217.5)	310.8 (202.0)	326.2 (221.8)	255.8 (413.2)	298.2 (230.3)	298.6 (228.4)	273.9 (178.1)	457.7 (609.9)
Female patients in panel, % (SD)	58.3 (15.0)	57.4 (15.1)	58.5 (15.3)	56.1 (18.8)	56.5 (14.3)	56.1 (14.5)	56.6 (14.4)	56.2 (15.5)

© 2022 Wang AZ et al. *JAMA Network Open.*

eTable 4 (continued). Characteristics of Currently-Exposed, Future-Exposed, and Never-Exposed Primary Care Physicians [a]

	**Breast Cancer Analysis**				**Colorectal Cancer Analysis**			
	**PCPs in Analysis**			**Never-Exposed PCPs** N=2260	**PCPs in Analysis**			**Never-Exposed PCPs** N=2788
	**All PCPs in Analysis** N=898[b]	**Currently-Exposed PCPs** N=567	**Future-Exposed PCPs** N=398		**All PCPs in Analysis** N=370 [b]	**Currently-Exposed PCPs** N=277	**Future-Exposed PCPs** N=183	
Patients enrolled in Medicaid in panel, % (SD)	14.6 (13.6)	14.4 (14.3)	14.0 (13.0)	22.2 (19.7)	14.9 (13.1)	14.3 (12.8)	14.2 (13.2)	20.6 (19.2)
Monthly patient visits, mean (SD)	68.9 (44.4) Female only: 42.5 (27.8)	70.4 (45.6) Female only: 42.7 (28.4)	64.5 (40.9) Female only: 39.4 (24.8)	43.6 (34.3) Female only: 24.6 (17.8)	63.7 (38.1)	63.2 (41.1)	55.7 (23.3)	37.7 (30.7)
Monthly screenings among patients who visited PCP, mean (SD)	Female only: 9.4 (6.6)	Female only: 9.2 (6.4)	Female only: 8.8 (6.3)	Female only: 4.5 (4.3)	4.5 (4.8)	4.4 (4.3)	3.5 (2.8)	2.2 (2.6)

[a] Table uses data from quarter-to-event -4 through -1 (pre-exposure period of one year). Patient panel sizes include only non-Medicare patients.

[b] N=898 unique PCPs in breast cancer analysis. N=370 unique PCPs in colorectal cancer analysis. PCPs in the stacked DD model can act as both currently-exposed and future-exposed PCPs at different time points. Thus, the column sums of currently- and future-exposed PCPs in the table are greater than the total number of individual exposed PCPs.

© 2022 Wang AZ et al. *JAMA Network Open.*

eTable 5. Falsification Test Results for Breast Cancer and Colorectal Cancer Analyses

**Falsification Tests:** Postexposure Change in Proportion of Patients Who Receive Cancer Screening Within Next Year				
	Breast Cancer Falsification Test		Colorectal Cancer Falsification Test	
	Absolute Change, Percentage Points (95% CI)	P-Value	Absolute Change, Percentage Points (95% CI)	P-Value
Pre-Exposure Period [Quarters -4 to -1]	0 [Reference]	NA	0 [Reference]	NA
Overall Post-Exposure Change	0.8 (-1.5 to 3.2)	0.49	-0.8 (-1.9 to 0.2)	0.13
Quarter 1	-0.9 (-3.8 to 2.0)	0.52	-0.6 (-1.9 to 0.7)	0.34
Quarter 2	-0.6 (-3.7 to 2.4)	0.69	0.0 (-1.3 to 1.4)	0.97
Quarter 3	0.2 (-3.0 to 3.3)	0.9	-0.3 (-1.8 to 1.3)	0.74
Quarter 4	1.7 (-1.9 to 5.2)	0.36	-0.3 (-1.9 to 1.4)	0.74

© 2022 Wang AZ et al. *JAMA Network Open.*

eTable 6. Robustness Tests: Results From Alternative Regression Specifications [a]

**Breast Cancer Analysis, Alternative Specifications:** Postexposure Change in Proportion of Patients Who Receive Cancer Screening Within Next Year		
	Absolute Change, Percentage Points (95% CI)	P-Value
**Main Analysis**	**4.5 (3.0 to 6.1)**	**<0.001**
**Seasonality indicators**: Calendar quarter (vs. calendar month)	4.6 (3.1 to 6.2)	< 0.001
**Time indicators**: Quarter (vs. year)	4.60 (3.0 to 6.3)	< 0.001
**Time indicators**: Month (vs. year)	4.60 (3.0 to 6.3)	< 0.001
**Include all PCPs** (vs. exclude PCPs in lowest fifth percentile panel size)	4.99 (3.4 to 6.6)	< 0.001
**Window of analysis**: -3 quarters to +3 quarters (vs. -4 to 4)	4.58 (3.2 to 5.9)	< 0.001
**Window of analysis**: -5 quarters to +5 quarters (vs. -4 to 4)	4.50 (2.6 to 6.4)	< 0.001
**Window of analysis**: -8 quarters to +8 quarters (vs. -4 to 4)	4.3 (2.7 to 5.8)	< 0.001
**Colorectal Cancer Analysis, Alternative Specifications:** Postexposure Change in Proportion of Patients Who Receive Cancer Screening Within Next Year		
	Absolute Change, Percentage Points (95% CI)	P-Value
**Main Analysis**	**1.3 (0.3 to 2.2)**	**0.01**
**Seasonality indicators**: Calendar quarter (vs. calendar month)	1.2 (0.2 to 2.2)	0.02
**Time indicators**: Quarter (vs. year)	1.2 (0.2 to 2.2)	0.02
**Time indicators**: Month (vs. year)	1.2 (0.2 to 2.2)	0.02
**Include all PCPs** (vs. exclude PCPs in lowest fifth percentile panel size)	1.1 (0.03 to 2.3)	0.04
**Window of analysis**: -3 quarters to +3 quarters (vs. -4 to 4)	1.3 (0.5 to 2.2)	0.003
**Window of analysis**: -5 quarters to +5 quarters (vs. -4 to 4)	1.1 (-0.01 to 2.2)	0.05
**Window of analysis**: -8 quarters to +8 quarters (vs. -4 to 4)	1.0 (0.0 to 2.0)	0.05

[a] For context, main analysis specifications are provided in parentheses for each analysis.

© 2022 Wang AZ et al. *JAMA Network Open.*

eTable 7. Sensitivity Analyses: Breast Cancer Analysis With Sequential Patient Age Restrictions

**Breast Cancer Analysis: Postexposure Change in Proportion of Patients Who Receive Cancer Screening Within Next Year<br><br>N=898 PCPs; Female Patients Only**								
	**Patients Aged 18-64 Years[a] (Main Analysis)**		**Patients Aged 35-64 Years[b]**		**Patients Aged 40-64 Years[c]**		**Patients Aged 50-64 Years[d]**	
	Mean Quarterly PCP Visits: 49.1 (SD=45.5) Median: 35.6		Mean Quarterly PCP Visits: 38.6 (SD=35.0) Median: 28.7		Mean Quarterly PCP Visits: 33.7 (SD=29.0) Median: 25.0		Mean Quarterly PCP Visits: 21.0 (SD=17.9) Median: 15.9	
	Absolute Change, Percentage Points (95% CI)	P-Value	Absolute Change, Percentage Points (95% CI)	P-Value	Absolute Change, Percentage Points (95% CI)	P-Value	Absolute Change, Percentage Points (95% CI)	P-Value
Pre-Exposure Period [Quarters -4 to -1]	0 [Reference]	NA	0 [Reference]	NA	0 [Reference]	NA	0 [Reference]	NA
Overall Post-Exposure Change	4.5 (3.0, 6.1)	< 0.001	2.6 (1.1, 4.2)	0.001	2.7 (1.0, 4.4)	0.002	1.6 (-0.4, 3.5)	0.12
Quarter 1	3.8 (2.2, 5.4)	< 0.001	2.0 (0.2, 3.7)	0.03	1.9 (0.1, 3.8)	0.04	0.8 (-1.4, 3.0)	0.48
Quarter 2	4.4 (2.5, 6.3)	< 0.001	2.2 (0.3, 4.2)	0.03	2.2 (0.0, 4.3)	0.04	1.6 (-0.9, 4.1)	0.21
Quarter 3	6.1 (3.8, 8.3)	< 0.001	2.9 (0.5, 5.2)	0.02	2.8 (0.2, 5.3)	0.03	1.3 (-1.6, 4.3)	0.39
Quarter 4	6.5 (4.2, 8.9)	< 0.001	3.8 (1.3, 6.3)	0.003	2.8 (0.2, 5.5)	0.04	1.0 (-2.1, 4.0)	0.53

[a] P = 0.44 for joint significance test of pre-exposure coefficients.
[b] P = 0.62 for joint significance test of pre-exposure coefficients.
[c] P = 0.42 for joint significance test of pre-exposure coefficients.
[d] P = 0.17 for joint significance test of pre-exposure coefficients.

© 2022 Wang AZ et al. *JAMA Network Open.*

eTable 8. Sensitivity Analyses: Colorectal Cancer Analysis With Sequential Patient Age Restrictions

Colorectal Cancer Analysis: Postexposure Change in Proportion of Patients Who Receive Cancer Screening Within Next Year<br><br>N=370 PCPs; Male and Female Patients								
	**Patients Aged 18-64 Years[a] (Main Analysis)**		**Patients Aged 35-64 Years[b]**		**Patients Aged 40-64 Years[c]**		**Patients Aged 50-64 Years[d]**	
	Mean Quarterly PCP Visits: 88.9 (SD=86.3) Median: 68.4		Mean Quarterly PCP Visits: 73.6 (SD=73.3) Median: 58.0		Mean Quarterly PCP Visits: 65.3 (SD=63.4) Median: 50.2		Mean Quarterly PCP Visits: 43.4 (SD=44.1) Median: 34.6	
	Absolute Change, Percentage Points (95% CI)	P-Value	Absolute Change, Percentage Points (95% CI)	P-Value	Absolute Change, Percentage Points (95% CI)	P-Value	Absolute Change, Percentage Points (95% CI)	P-Value
Pre-Exposure Period [Quarters -4 to -1]	0 [Reference]	NA	0 [Reference]	NA	0 [Reference]	NA	0 [Reference]	NA
Overall Post-Exposure Change	1.3 (0.3, 2.2)	0.01	0.9 (-0.2, 2.0)	0.09	0.3 (-0.8, 1.5)	0.57	-0.2 (-1.6, 1.3)	0.82
Quarter 1	1.4 (0.3, 2.5)	0.02	0.7 (-0.4, 1.9)	0.20	0.6 (-0.6, 1.8)	0.32	-0.2 (-1.7, 1.3)	0.75
Quarter 2	2.2 (0.9, 3.4)	0.001	1.7 (0.3, 3.0)	0.02	1.5 (0.1, 2.9)	0.03	0.5 (-1.3, 2.3)	0.59
Quarter 3	2.0 (0.5, 3.4)	0.01	1.6 (0.0, 3.1)	0.04	0.8 (-0.7, 2.3)	0.32	-0.1 (-2.1, 1.8)	0.88
Quarter 4	2.1 (0.8, 3.4)	0.001	1.3 (-0.4, 2.9)	0.13	1.0 (0.7, 2.7)	0.25	-0.6 (-2.7, 1.5)	0.59

[a] P = 0.13 for joint significance test of pre-exposure coefficients.
[b] P = 0.22 for joint significance test of pre-exposure coefficients.
[c] P = 0.64 for joint significance test of pre-exposure coefficients.
[d] P = 0.47 for joint significance test of pre-exposure coefficients.

© 2022 Wang AZ et al. *JAMA Network Open.*

eTable 9. Sensitivity Analysis: No Carcinoma In Situ Diagnoses[a]

Postexposure Change in Proportion of Patients Who Receive Cancer Screening Within Next Year – No DCIS/CIS Diagnoses						
	Breast Cancer Analysis			Colorectal Cancer Analysis		
	Absolute Change, Percentage Points	95% Confidence Interval	P-Value	Absolute Change, Percentage Points	95% Confidence Interval	P-Value
Pre-Exposure Period [Quarters -4 to -1]	0 [Reference]	NA	NA	0 [Reference]	NA	NA
Overall Post-Exposure Change	4.6	[3.0, 6.1]	< 0.001	1.3	[0.3, 2.2]	0.01
Quarter 1	3.7	[2.1, 5.3]	< 0.001	1.5	[0.4, 2.7]	0.01
Quarter 2	4.2	[2.4, 6.1]	< 0.001	2.0	0.9, 3.2]	< 0.001
Quarter 3	5.8	[3.6, 8.0]	< 0.001	1.8	[0.4, 3.3]	0.01
Quarter 4	6.8	[4.5, 9.2]	< 0.001	2.0	[0.7, 3.3]	0.003

[a]This sensitivity analysis excludes diagnosis (ICD) codes for carcinoma in situ. Breast cancer analysis: P = 0.38 for joint significance test of pre-exposure coefficients. Colorectal cancer analysis: P = 0.13 for joint significance test of pre-exposure coefficients.

© 2022 Wang AZ et al. *JAMA Network Open.*

eTable 10. Sensitivity Analysis: No Exposures Followed by a Subsequent Exposure Within the Same Year[a]

Postexposure Change in Proportion of Patients Who Receive Cancer Screening Within Next Year						
	Breast Cancer Analysis			Colorectal Cancer Analysis		
	Absolute Change, Percentage Points	95% Confidence Interval	P-Value	Absolute Change, Percentage Points	95% Confidence Interval	P-Value
Pre-Exposure Period [Quarters -4 to -1]	0 [Reference]	NA	NA	0 [Reference]	NA	NA
Overall Post-Exposure Change	4.6	[3.1, 6.2]	< 0.001	1.2	[0.2, 2.2]	0.02
Quarter 1	3.9	[2.2, 5.5]	< 0.001	1.3	[0.2, 2.4]	0.02
Quarter 2	4.5	[2.6, 6.3]	< 0.001	2.2	[0.9, 3.4]	0.001
Quarter 3	6.2	[3.9, 8.4]	< 0.001	1.7	[0.2, 3.3]	0.03
Quarter 4	6.7	[4.3,9.0]	< 0.001	2.0	[0.6, 3.3]	0.004

[a]This sensitivity analysis excludes PCPs' exposures to new cancer if the same PCP is exposed to another new cancer diagnosis within the same year of the first exposure. Breast cancer analysis: P = 0.62 for joint significance test of pre-exposure coefficients. Colorectal cancer analysis: P = 0.12 for joint significance test of pre-exposure coefficients.

© 2022 Wang AZ et al. *JAMA Network Open.*

eTable 11. Sensitivity Analysis: PCPs With Two or Fewer Total Exposures[a]

Postexposure Change in Proportion of Patients Who Receive Cancer Screening Within Next Year						
	Breast Cancer Analysis			Colorectal Cancer Analysis		
	Absolute Change, Percentage Points	95% Confidence Interval	P-Value	Absolute Change, Percentage Points	95% Confidence Interval	P-Value
Pre-Exposure Period [Quarters -4 to -1]	0 [Reference]	NA	NA	0 [Reference]	NA	NA
Overall Post-Exposure Change	5.3	[3.5, 7.1]	< 0.001	1.2	[0.2, 2.2]	0.02
Quarter 1	4.6	[2.7, 6.5]	< 0.001	1.3	[0.2, 2.5]	0.02
Quarter 2	5.5	[3.3, 7.6]	< 0.001	2.1	[0.9, 3.4]	0.001
Quarter 3	6.3	[3.7, 8.9]	< 0.001	1.7	[0.2, 3.3]	0.03
Quarter 4	7.2	[4.5, 9.8]	< 0.001	19	[0.6, 3.3]	0.01

[a]This sensitivity analysis excludes PCPs with greater than two total exposures over the entire exposure period. Breast cancer analysis: P = 0.54 for joint significance test of pre-exposure coefficients. Colorectal cancer analysis: P = 0.11 for joint significance test of pre-exposure coefficients.

© 2022 Wang AZ et al. *JAMA Network Open.*

eTable 12. Sensitivity Analysis: 8-Quarter Analytical Window

Postexposure Change in Proportion of Patients Who Receive Cancer Screening Within Next Year						
	Breast Cancer Analysis			Colorectal Cancer Analysis		
	Absolute Change, Percentage Points	95% Confidence Interval	P-Value	Absolute Change, Percentage Points	95% Confidence Interval	P-Value
Pre-Exposure Period [Quarters -8 to -1]	0 [Reference]	NA	NA	0 [Reference]	NA	NA
Overall Post-Exposure Change	4.3	[2.7, 5.8]	< 0.001	1.0	[0.0, 2.0]	0.05
Quarter 1	3.9	[2.3, 5.5]	< 0.001	1.4	[0.2, 2.5]	0.02
Quarter 2	4.3	[2.4, 6.1]	< 0.001	2.2	[1.0, 3.4]	0.001
Quarter 3	6.0	[3.8, 8.3]	< 0.001	2.0	[0.6, 3.4]	0.01
Quarter 4	6.7	[4.4, 9.0]	< 0.001	2.1	[0.9, 3.4]	0.001
Quarter 5	7.2	[4.8, 9.7]	< 0.001	2.2	[0.8, 3.5]	0.002
Quarter 6	5.9	[3.3, 8.6]	< 0.001	2.2	[0.8, 3.6]	0.002
Quarter 7	5.6	[2.9, 8.4]	< 0.001	2.5	[1.0, 3.9]	0.001
Quarter 8	5.6	[2.9, 8.3]	< 0.001	3.1	[1.4, 4.9]	< 0.001

[a]This sensitivity analysis uses a +/- 8-quarter window for the analysis instead of a +/- 4 quarter window. Breast cancer analysis: P = 0.53 for joint significance test of pre-exposure coefficients. Colorectal cancer analysis: P = 0.21 for joint significance test of pre-exposure coefficients.

© 2022 Wang AZ et al. *JAMA Network Open.*

eTable 13. Additional Analysis: Colorectal Cancer Analysis, Stratified by Categories of Cancer Screening Modality [a]

Postexposure Change in Proportion of Patients Who Receive Cancer Screening Within Next Year						
	Colonoscopy		FOBT/FIT		Sigmoidoscopy	
	Absolute Change, Percentage Points (95% CI)	P-Value	Absolute Change, Percentage Points (95% CI)	P-Value	Absolute Change, Percentage Points (95% CI)	P-Value
Pre-Exposure Period [Quarters -4 to -1]	0 [Reference]	NA	0 [Reference]	NA	0 [Reference]	NA
Overall Post-Exposure Change	0.6 (-0.3 to 1.4)	0.20	0.7 (0.0 to 1.3)	0.04	0.0 (-0.1 to 0.2)	0.56
Quarter 1	0. 9 (-0.2 to 1.9)	0.10	0.5 (-0.1 to 1.2)	0.11	0.0 (-0.1 to 0.1)	0.97
Quarter 2	1.0 (0.1 to 1.9)	0.03	1.0 (0.2 to 1.8)	0.02	0.0 (-0.1 to 0.2)	0.60
Quarter 3	1.1 (-0.2 to 2.4)	0.11	0.8 (-0.1 to 1.7)	0.10	0.1 (-0.1 to 0.3)	0.34
Quarter 4	1.2 (0.1 to 2.3)	0.03	0.7 (-0.3 to 1.7)	0.16	0.2 (0.0 to 0.4)	0.10

[a] Colorectal cancer analyses were stratified into three cancer modality categories: 1) “Colonoscopy:” colonoscopy only; 2) “FOBT/FIT:” fecal occult blood testing (FOBT), fecal immunochemical testing (FIT), and/or multitarget stool DNA testing; and 3) “Sigmoidoscopy:” sigmoidoscopy only. Colonoscopy analysis: P = 0.12 for joint significance test of pre-exposure coefficients. FOBT/FIT analysis: P = 0.68 for joint significance test of pre-exposure coefficients. Sigmoidoscopy analysis: P = 0.51 for joint significance test of pre-exposure coefficients.

© 2022 Wang AZ et al. *JAMA Network Open.*

eReferences

1. Nattinger AB, Laud PW, Bajorunaite R, Sparapani RA, Freeman JL. An algorithm for the use of Medicare claims data to identify women with incident breast cancer. *Health Serv Res*. 2004;39(6 Pt 1):1733-1749. doi:10.1111/j.1475-6773.2004.00315.x

2. Rolnick SJ, Hart G, Barton MB, et al. Comparing breast cancer case identification using HMO computerized diagnostic data and SEER data. *Am J Manag Care*. 2004;10(4):257-262.

3. Quantin C, Benzenine E, Hägi M, et al. Estimation of National Colorectal-Cancer Incidence Using Claims Databases. *Journal of Cancer Epidemiology*. 2012;2012:1-7. doi:10.1155/2012/298369

4. Deshpande M, Li Y. Who Is Screened Out? Application Costs and the Targeting of Disability Programs. *American Economic Journal: Economic Policy*. 2019;11(4):213-248. doi:10.1257/pol.20180076

5. Fadlon I, Nielsen TH. Family Health Behaviors. *American Economic Review*. 2019;109(9):3162-3191. doi:10.1257/aer.20171993

6. Han D, Khadka A, McConnell M, Cohen J. Association of Unexpected Newborn Deaths With Changes in Obstetric and Neonatal Process of Care. *JAMA Netw Open*. 2020;3(12):e2024589. doi:10.1001/jamanetworkopen.2020.24589

© 2022 Wang AZ et al. *JAMA Network Open.*
